# S Mi-Annual Meeting of Erie County Medical Society

**Published:** 1871-06

**Authors:** 


### Semi-Annual Meeting of Erie County Medical Society.

This society held its semi-annual meeting the second Tuesday in June; a large number of the members in attendance.

The admission of new members, report of committee on fee-bill, and report of committee appointed at the annual meeting to investigate charges made by Dr. Edward Storck, against Dr. Otto Burger, were the main items of business.

The report of the committee was, in effect, that the charges of consulting with irregular physicians, and of unprofessional conduct, were sustained.

Dr. Storck moved that Dr. Otto Burger be expelled from the society. Dr. Burger introduced affidavits intended as contradictory of the charges, and most of the members indulged in expression of opinions upon the general question involved, or upon the collateral questions supposed to be worthy of discussion.

As this society have heretofore had some experience in expelling members and refusing applicants for membership, slight differences of opinion were manifest as to the legal rights of the society, and the regularity of its own action. After a lengthy and very free expression of opinion, occupying most of the day Tuesday, and all the time of an adjourned meeting one week later, the final ballot was taken, resulting in a vote for expulsion by a very large majority.

